# Supplementary material for: Effects of nutritional supplements on the re-infection rate of soil-transmitted helminths in school-age children: A systematic review and meta-analysis
Source: PLoS One. 2020 Aug 13;15(8):e0237112. doi: 10.1371/journal.pone.0237112 (PMC7425883; doi:10.1371/journal.pone.0237112)
Supplement: S1 Appendix — (DOCX) [file pone.0237112.s002.docx]

**Appendix 1**

**Database: Ovid MEDLINE**(R) and Epub Ahead of Print, In-Process & Other Non-Indexed Citations and Daily <1946 to April 17, 2019,>

Search Strategy:

--------------------------------------------------------------------------------

1 (proteins or vitamin E or vitamin C or Vitamin B6 or vitamin A or iron or zinc or folate or calcium or iodine or potassium or micronutrient or powders or food).mp. (4204334)

2 Minerals/ (22269)

3 Vitamins/ (29891)

4 Vitamin D/ (31994)

5 1 or 2 or 3 or 4 (4242537)

6 Infection/ (37271)

7 (reinfection or recurrence or reoccurrence).mp. (458428)

8 Soil/ (49796)

9 (Helminths or Trichuris or Ascariasis or Ascaridida or Necatoriasis or ancylostomiasis or Ascaris or Trichuriasis or ancylostoma or ancylostomatoidea or necator).mp. (27370)

10 6 or 7 (494846)

11 5 or 10 (4702313)

12 8 or 9 (76340)

13 11 and 12 (14248)

14 limit 13 to clinical trial, all (117)

**Database: Embase<1974 to 2019 April 17>**

Search Strategy:

--------------------------------------------------------------------------------

1 (proteins or vitamin E or vitamin C or Vitamin B6 or vitamin A or iron or zinc or folate or calcium or iodine or potassium or micronutrient or powders or food).mp. (3443988)

2 mineral/ (38291)

3 vitamin/ (37496)

4 vitamin D/ (68675)

5 1 or 2 or 3 or 4 (3507661)

6 infection/ (301048)

7 (reinfection or recurrence or reoccurrence).mp. (533152)

8 soil/ (55199)

9 (Helminths or Trichuris or Ascariasis or Ascaridida or Necatoriasis or ancylostomiasis or Ascaris or Trichuriasis or ancylostoma or ancylostomatoidea or necator).mp. (23338)

10 6 or 7 (823245)

11 5 or 10 (4274421)

12 8 or 9 (77748)

13 11 and 12 (16534)

14 limit 13 to (clinical trial or randomized controlled trial or controlled clinical trial or multicentre study or phase 1 clinical trial or phase 2 clinical trial or phase 3 clinical trial or phase 4 clinical trial) (197)

**Appendix 3: Sensitivity Analysis**

Figure i: Sensitivity analysis showing significant reduction of *Ascaris lumbroicoides* reinfection with iron supplements rate when fixed effect model is used rather than random effect model. Outcomes represent prevalence rates (%) of infection at follow-up

Figure j: Sensitivity analysis showing significant reduction of *Ascaris lumbroicoides* reinfection rate with multimicronutrients when fixed effect model is used rather than random effect model. Outcomes represent prevalence rates (%) of infection at follow-up

Figure k: Sensitivity analysis showing significant reduction of hookworm reinfection rate with multimicronutrients when fixed effect model is used rather than random effect model. Outcomes represent prevalence rates (%) of infection at follow-up
