# Supplementary material for: Effects of nutritional supplements on the re-infection rate of soil-transmitted helminths in school-age children: A systematic review and meta-analysis
Source: PLoS One. 2020 Aug 13;15(8):e0237112. doi: 10.1371/journal.pone.0237112 (PMC7425883; doi:10.1371/journal.pone.0237112)
Supplement: Attachment — Submitted filename: Plosone_may2020.docx [file pone.0237112.s003.docx]

Page 4: line 72-73; For the last part of the sentence: ‘and walking barefoot on contaminated soil’ is only the case for hookworm not for the other helminths

Page 4: line 84: ‘The health consequences of STH infections may plunge further the children from low-income neighborhoods into poverty since infected children possibly have worse school performance’

I think it would be better to write: The health consequences of STH infections may ~~the~~ plunge children further from low-income neighborhoods into poverty since infected children possibly have worse school performance

Page 6: line 136: the secondary outcome: interesting that the authors also want to investigate this. However how does this relate to the research question? I would suggest to either add this as a sub question in the introduction or explain better how this related to the RQ.

Also this secondary outcome measure is not mentioned in the rest of the methods section.

Page 7: it would be good to add the exact search terms that were used and also the dates that the searches were performed.

Page 9, figure 1:

- ‘additional records” ; i find this is a bit vague. else where in the manuscript (page 13) the authors state that these record were identified from the reference list from the selected papers. It would be better to be consistent.
- Full text excluded with reasons; this is also too vague. Please reformulate.

Page 12: line 276; the selection process is described to superficially. It would be good to elaborate on this process.

Page 23: line 347: ‘the three studies were not able to prove’ . Think a better formulation would be were not able to report. Prove is too strong in my opinion.

Page 27; line 415 multi-micronutrients; this is the only meta-analysis which is statistically significant, however this is not mentioned. I wonder why? Also this meta-analysis (even though based on 2 studies) shows that MM’s might have adverse effects. I think it would be good to give important this finding more attention. Please also elaborate on this important finding in the discussion.

Page 28: line 428: effect on school productivity.

This outcome is not mentioned before. Please also elaborate on this in the methods.

For the discussion I would recommend to;

- Include the value of observationational (longitudinal) studies, as eventhough they may be more prone to confounding, they do often have longer followup time and may thus provide more evidence.
- Give more attention to the possibility of unfavourable effect of MM supplementation.
